# Prediction of myocardial ischemia in coronary heart disease patients using a CCTA–Based radiomic nomogram

**DOI:** 10.3389/fcvm.2023.1024773

**Published:** 2023-01-19

**Authors:** You-Chang Yang, Yang Dou, Zhi-Wei Wang, Ruo-Han Yin, Chang-Jie Pan, Shao-Feng Duan, Xiao-Qiang Tang

**Affiliations:** ^1^Department of Radiology, Qilu Hospital (Qingdao), Cheeloo College of Medicine, Shandong University, Shandong, China; ^2^Graduate School of Dalian Medical University, Dalian Medical University, Dalian, China; ^3^Department of Radiology, The Affiliated Changzhou No. 2 People’s Hospital of Nanjing Medical University, Changzhou, China; ^4^GE Healthcare, Precision Health Institution, Shanghai, China

**Keywords:** X-ray computed, diagnosis, differential, radiomics, myocardial ischemia

## Abstract

**Objective:**

The present study aimed to predict myocardial ischemia in coronary heart disease (CHD) patients based on the radiologic features of coronary computed tomography angiography (CCTA) combined with clinical factors.

**Methods:**

The imaging and clinical data of 110 patients who underwent CCTA scan before DSA or FFR examination in Changzhou Second People’s Hospital, Nanjing Medical University (90 patients), and The First Affiliated Hospital of Soochow University (20 patients) from March 2018 to January 2022 were retrospectively analyzed. According to the digital subtraction angiography (DSA) and fractional flow reserve (FFR) results, all patients were assigned to myocardial ischemia (*n* = 58) and normal myocardial blood supply (*n* = 52) groups. All patients were further categorized into training (*n* = 64) and internal validation (*n* = 26) sets at a ratio of 7:3, and the patients from second site were used as external validation. Clinical indicators of patients were collected, the left ventricular myocardium were segmented from CCTA images using CQK software, and the radiomics features were extracted using pyradiomics software. Independent prediction models and combined prediction models were established. The predictive performance of the model was assessed by calibration curve analysis, receiver operating characteristic (ROC) curve and decision curve analysis.

**Results:**

The combined model consisted of one important clinical factor and eight selected radiomic features. The area under the ROC curve (AUC) of radiomic model was 0.826 in training set, and 0.744 in the internal validation set. For the combined model, the AUCs were 0.873, 0.810, 0.800 in the training, internal validation, and external validation sets, respectively. The calibration curves demonstrated that the probability of myocardial ischemia predicted by the combined model was in good agreement with the observed values in both training and validation sets. The decision curve was within the threshold range of 0.1–1, and the clinical value of nomogram was higher than that of clinical model.

**Conclusion:**

The radiomic characteristics of CCTA combined with clinical factors have a good clinical value in predicting myocardial ischemia in CHD patients.

## 1. Introduction

Coronary heart disease (CHD) is a frequent disease that seriously threatens people’s health. Coronary artery disease (CAD) is the main cause of cardiovascular disease death worldwide (1–3). Therefore, early detection and timely interventional treatment are very important for these patients. Patients with CHD mainly present with stable ischemic heart disease. The indication for surgery in these patients is not only the degree of coronary artery stenosis, but also the severity of myocardial ischemia. Therefore, accurate assessment of patients with myocardial ischemia will affect the clinician’s choice of treatment for these patients. Coronary computed tomography Angiography (CCTA) is currently the first choice for screening CHD patients, which is sensitive to the nature of plaque and the degree of stenosis (4, 5), but cannot directly obtain information about myocardial ischemia (6). At present, the gold standard models for the diagnosis of myocardial ischemia include fractional flow reserve (FFR), positron emission tomography (PET), and single-photon emission computed tomography (SPECT) (7). However, these detection methods are invasive and expensive, so they are not commonly applied in the clinic. Therefore, it is important to seek an economical and non-invasive examination method to evaluate myocardial ischemia.

Radiomics is a new quantitative imaging technique that is commonly employed in medical research because of its non-invasive nature and cost-effectiveness. In recent years, many studies have shown that machine learning-based radiomic algorithm can be employed to capture hidden features in imaging to assist clinicians in accurately diagnosing patients and predicting treatment responses (8–10). At present, radiomic analysis can be performed for the diagnosis of cardiomyopathy, myocardial infarction, and arrhythmia (11–13). Some studies have confirmed that radiomics can predict myocardial ischemia, but these experiments are only based on CCTA images and do not combine with other clinical indicators (14–19).

Therefore, the present study aimed to develop a diagnostic model for predicting myocardial ischemia by integrating radiomic features and clinical data.

## 2. Materials and methods

**FIGURE 1:**
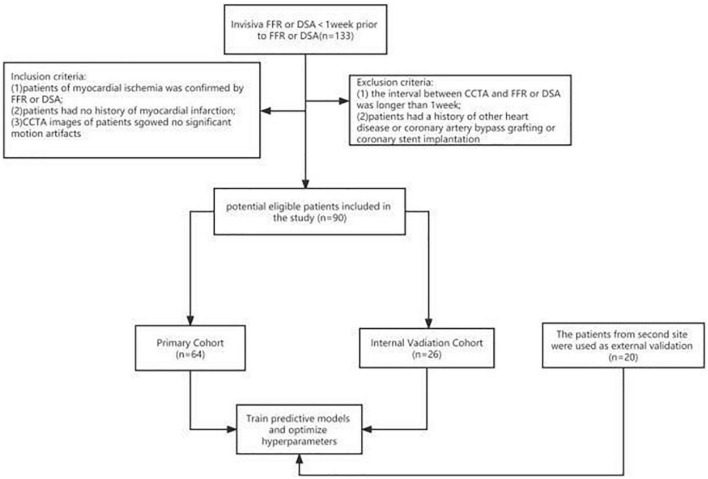
Flowsheet of patient selection.

This retrospective study was approved by the Ethics Committee of Changzhou Second People’s Hospital, Nanjing Medical University. The inclusion criteria for patients were as follows: (i) patients with myocardial ischemia were confirmed by FFR ≤ 0.8 or DSA ≥ 90%; (ii) patients had no history of myocardial infarction; and (iii) CCTA images of patients showed no significant motion artifacts. The exclusion criteria were as follows: (i) the interval between CCTA and FFR or DSA was longer than 1 week; and (ii) patients had a history of other cardiac diseases or coronary artery bypass grafting or coronary stent implantation. Ultimately, 110 patients were enrolled from 2 sites in the study. The process of patient selection is illustrated in Figure 1.

### 2.1. Patient recruitment

All included patients from site 1 were randomly divided into the training (*n* = 64) and internal validation (*n* = 26) sets at a ratio of 7:3. The patients from site 2 were assigned to the external validation set (*n* = 20). In the training set, 34 patients had myocardial ischemia and the remaining 30 patients had normal myocardial blood supply, as verified by digital subtraction angiography (DSA) and fractional flow reserve (FFR) assessment. In the internal validation set, 14 patients had myocardial ischemia, and the remaining 12 patients had normal myocardial blood supply. In the external validation set, 10 patients had myocardial ischemia, and the remaining 10 patients had normal myocardial blood supply.

### 2.2. CT image acquisition

The CT scans were performed using a 256-detector CT (GE Revolution CT, GE Healthcare, America), dual source CT (SOMATOM Definition, Siemens Healthineers, Germany), and dual source CT (SOMATOM Force, Siemens Healthineers, Germany). The scan range of cardiac examination was from the thoracic entrance to the diaphragmatic surface of the heart (20). Table 1 shows the scanning parameters used in this study.

**TABLE 1:** The scanning parameters of coronary computed tomography angiography (CCTA).

Parameter	Value
Tube current	Automatic modulation
Tube voltage	120 kVP
Slice thickness	0.75 mm
Collimation	1.0 mm
Reconstruction increment	0.5 mm
Convolution kernel	B26 f
The injection rate of contrast agent	6 ml/s

### 2.3. Image segmentation and feature extraction

Two radiologists (R1 and R2) with 5 years of work experience in the cardiovascular group imported the CCTA images into CQK software for automatic myocardial segmentation using a pre-trained V-NET model, the left ventricular myocardium were segmented and then visually checked and manually modified by the radiologists. R1 took 1 week to perform two operations on the images over an interval, and R2 operated only once. Ultimately, one patient’s image corresponded to three processing results. Finally, the extraction of myocardial radiomics features was performed using pyradiomics software (version 3.0.1^1^). Specifically, resampling the image voxel size with 1*1*1, discretizing the CT gray value using binWidth 25.

### 2.4. Feature selection and model establishment

**FIGURE 2:**
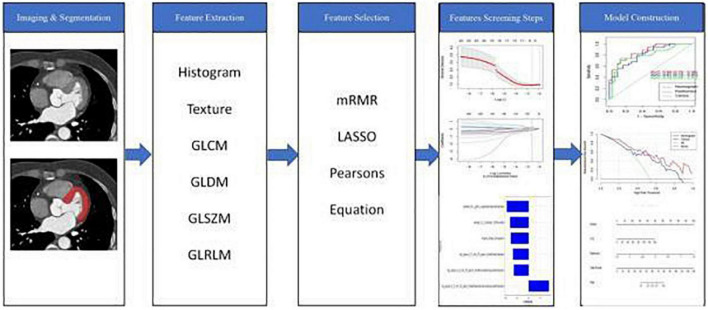
Workflow of the radiomic analysis.

**FIGURE 3:**
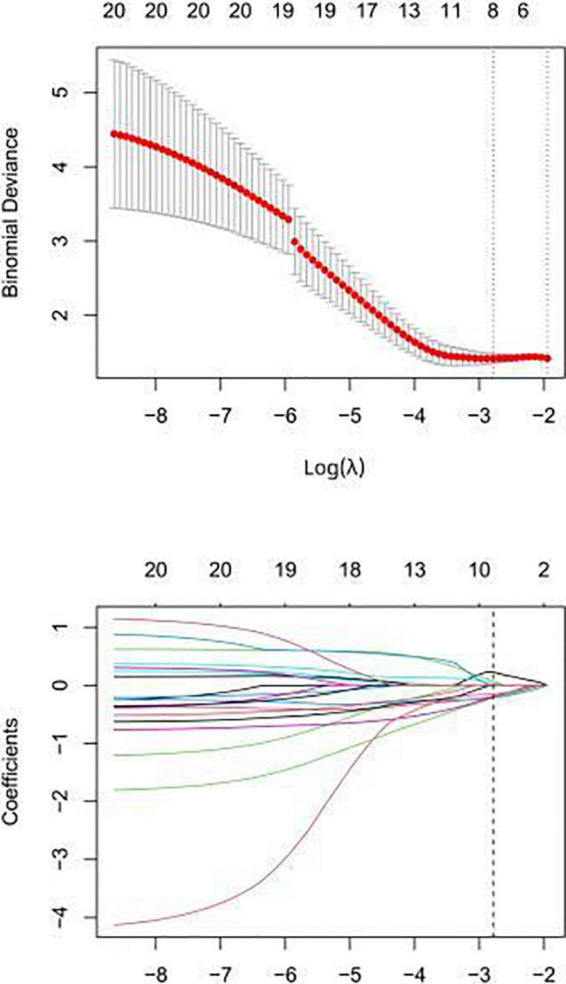
In the lasso regression model, the folding cross test is used to select the optimal value of the hyperparameter λ, and the lowest is the feature that best matches the true result.

A total of 1,218 radiomic features were extracted, as shown in Figure 2. Pearson correlation coefficient was used to eliminate redundant radiomic features and select the best feature among the remaining radiomic features. First, radiomic features with ICCs > 0.75 were selected to ensure inter-observer consistency and intra-observer consistency. Secondly, the maximum redundancy minimum correlation (mRMR) and lasso regression algorithms were employed to reduce the dimension of the features. The 10-fold cross-test method was used to screen the optimal hyperparameter λ value of the lasso regression model and construct the radiomics model (Figure 3).

Univariate logistic regression analysis was conducted to explore whether there was a significant difference in the characteristics between the two groups. Then, multivariable logistic regression was used to analyze those clinical factors and establish a combined clinical-radiological model and predictive nomogram. Besides, a predictive model was developed based on the selected clinical parameters without radiomics scores.

### 2.5. Model validation

ROC analyses were performed to evaluate the performance of radiomics model, clinical model and combined model in training and internal validation sets. The agreements between the observed ischemia probabilities and model predictive ischemia probabilities in training and internal validation sets were assessed by the calibration curves. The combined model’s clinical usefulness was studied using decision curve analyses. In order to validate the combined model’s generalization, we also evaluate the combined model in the external validation set.

### 2.6. Statistical analysis

In this study, SPSS 25.0 and R 4.0.2^2^ were employed for statistical analyses. Measurement data were assessed by Shapiro–Wilk normality, and those with normal distribution were presented as $\bar{X}$ ± *S.* Statistical differences between the two groups were compared by independent sample *t*-test. The enumeration data were presented as the number of cases, and the comparison between the two groups was carried out with chi-square test. Univariate and multivariate logistic regression methods were used to select clinical factors and included in the clinical factor prediction model. The differential diagnostic performance of radiomic model and combined diagnostic model was evaluated by ROC curve, and the area under curve (AUC), 95% CI, accuracy, specificity, and sensitivity were calculated. Statistical difference in AUC between pairwise models was compared by DeLong test.

## 3. Results

### 3.1. General patient information

Ninety patients were included in this study, including 39 females and 51 males. The general data of the patients and independent clinical factors (multiple logistic regression analysis) are presented in Tables 2, 3.

**TABLE 2:** General patient information.

	Training group (*n* = 64)	Internal validation group (*n* = 26)	External validation group (*n* = 20)	*P*-value
Age	65 (10)	64 (12)	68 (11)	0.417
Gender				0.843
Male	46 (71.9%)	14 (53.8%)	15 (75.0%)	
Female	18 (28.1%)	12 (46.2%)	5 (25.0%)	
Hypertension				0.884
Yes	46 (71.9%)	21 (80.8%)	15 (75.0%)	
No	18 (28.1%)	5 (19.2%)	5 (25.0%)	
Hyperlipidemia				0.809
Yes	32 (50.0%)	15 (57.7%)	7 (35.0%)	
No	32 (50.0%)	11 (42.3%)	13 (65.0%)	
Diabetes				0.796
Yes	28 (43.8%)	10 (38.5%)	9 (45.0%)	
No	36 (56.3%)	16 (61.5%)	11 (55.0%)	
Drinking				0.086
Yes	10 (15.6%)	5 (19.2%)	3 (15.0%)	
No	54 (84.4%)	21 (80.8%)	17 (85.0%)	
Smoking				0.939
Yes	25 (39.1%)	11 (42.3%)	7 (35.0%)	
No	39 (60.9%)	15 (57.7%)	13 (65.0%)	

**TABLE 3:** Clinical factors.

			95% CI	
**Factors**	**Odds ratio**	**P-value**	**Lower**	**Upper**
Gensini score	1.04	0.014879	1.01	1.08
Radscore	46.48	0.001516	4.33	498.45

CI, confidence interval.

### 3.2. Feature selection and radiomic model construction

**FIGURE 4:**
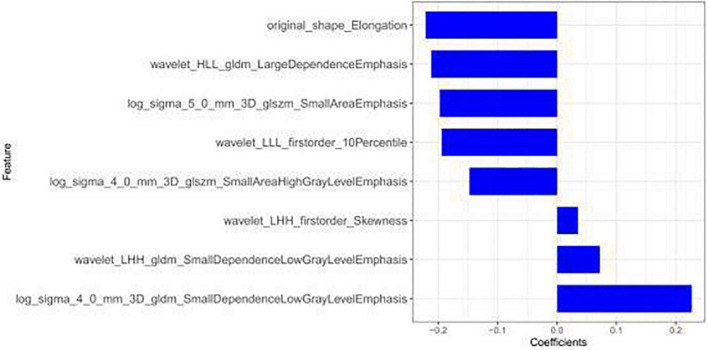
Eight radiomic features and their corresponding coefficients remained after screening.

Feature overfitting was avoided by reducing the dimension of the features. Firstly, among the 322 features extracted from CCTA images, features with ICCs > 0.75 were considered to have good inter-observer consistency and intra-observer consistency. Then, in order to remove the irrelevant and redundant features and screen out the features with good consistency, the mRMR method was used for processing, and the number of features was reduced to 30. Finally, the subset was optimized by Lasso regression analysis (Figure 3). The selected features are displayed in Figure 4. The rad-score was determined as follows:

Radscore = 0.227*log_sigma_4_0_mm_3D_gldm_Small DependenceLowGrayLevelEmphasis − 0.194*wavelet_LLL_first order_ 10 Percentile − 0.212*wavelet_HLL_gldm_LargeDependence Emphasis + 0.035*wavelet_LHH_firstorder_Skewness − 0.197*log_ sigma_5_0_mm_3D_glszm_SmallAreaEmphasis − 0.148*log_sigma_ 4_0_mm_3D_glszm_SmallAreaHighGrayLevelEmphasis − 0.221* original_shape_Elongation + 0.072*wavelet_LHH_gldm_Small DependenceLowGrayLevelEmphasis + 0.138.

**FIGURE 5:**
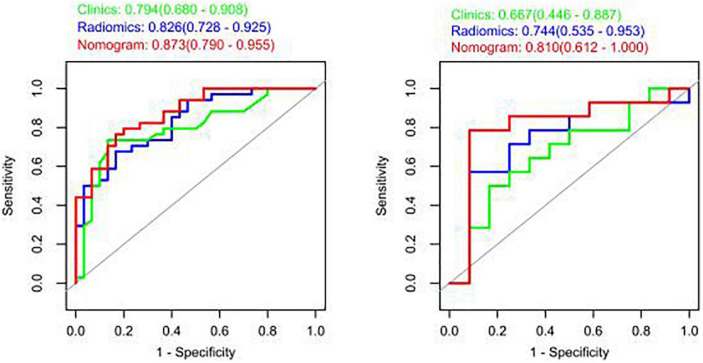
Receiver operating characteristic (ROC) curves of the radiomic, clinical, and integrated models.

The model constructed by the selected eight radiomics features exhibited good discrimination ability, with AUC values of 0.826, 0.744 in the training and internal validation sets, respectively (Figure 5). The accuracy, sensitivity, and specificity of this model are presented in Table 4.

**TABLE 4:** Performance of the models.

Model	Cut-off		Accuracy (95% CI)	Sensitivity	Specificity	PPV	NPV
Radiomics	0.220	Training	0.75 [0.63; 0.85]	0.68	0.83	0.82	0.69
		Internal validation	0.65 [0.44; 0.83]	0.57	0.75	0.73	0.60
Clinical	−0.055	Training	0.80 [0.68; 0.89]	0.74	0.87	0.86	0.74
		Internal validation	0.65 [0.44; 0.83]	0.57	0.75	0.72	0.60
Nomogram	0.202	Training	0.80 [0.68; 0.89]	0.76	0.83	0.84	0.76
		Internal validation	0.77 [0.56; 0.910]	0.79	0.75	0.79	0.75
		External validation	0.65 [0.41; 0.85]	0.50	0.80	0.71	0.62

CI, confidence interval; PPV, positive-predictive value; NPV, negative-predictive value.

### 3.3. Clinical value of the nomogram

**FIGURE 6:**
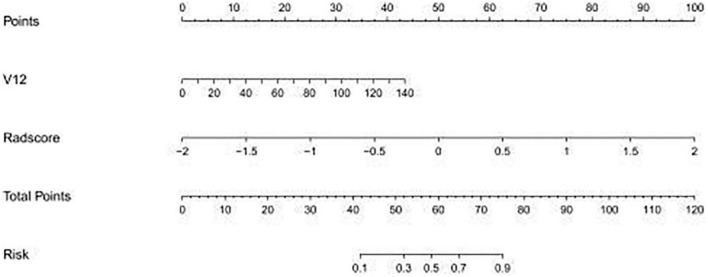
The established clinical-radiomic nomogram for predicting myocardial ischemia. V12, Gensini score.

**FIGURE 7:**
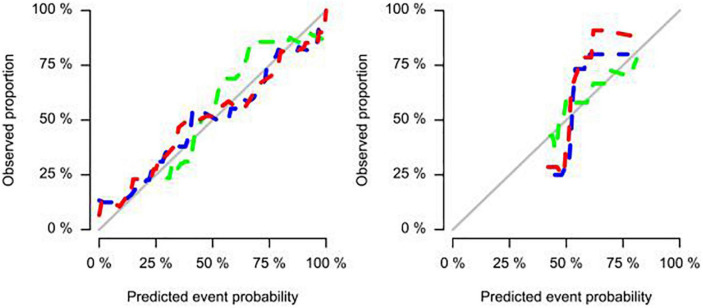
Calibration curves of the integrated nomogram for predicting myocardial ischemia in both training, internal validation, and external validation sets.

The study showed that the combined model based on CCTA imaging features combined with key clinical factors was significantly superior to the prediction efficiency of the clinical model, and the AUC value was increased from 0.67 to 0.81 (Table 4 and Figure 5). Compared to the clinical model alone, the combination of clinical and radiomic features markedly enhanced the prediction of myocardial ischemia. The Delong test also indicated that the combined nomogram (AUC of 0.810 in the internal validation set) was a better predictor of myocardial ischemia than radiomics alone. A clinical-radiomic nomogram was constructed to visualize the models (Figure 6). The calibration curves of the integrated nomogram for predicting myocardial ischemia showed good agreement in the training and validation sets. Good calibration curves were detected, and the HL test indicated goodness of fit to the data (Figure 7). The nomo-score was calculated as follows:


$$\mathrm{Nomoscore}={\mathrm{V12}}^{*}\,0.041+{\mathrm{Radscore}}^{*}\,3.291-1.097$$

**FIGURE 8:**
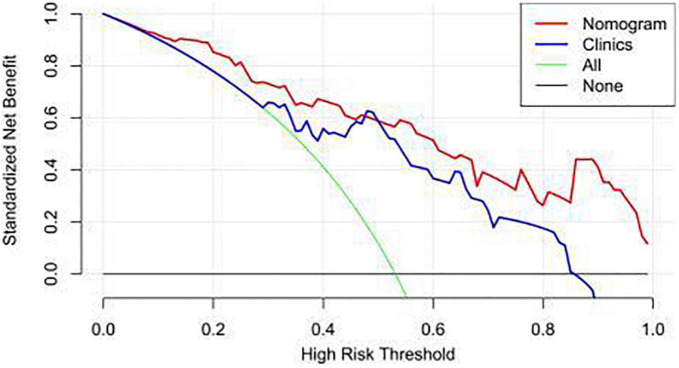
Decision curve analysis.

By adding radiomic features, the combined clinical-radiomics DCAs had better clinical utility, particularly the clinical DCA, indicating that the nomogram is robust clinical tool for predicting myocardial ischemia. The decision curve was within the threshold range of 0.1–1, and the clinical value of the nomogram was higher than that of the clinical model (Figure 8).

## 4. Discussion

To detect myocardial ischemia as early as possible, this study systematically analyzed the differences in clinically independent risk factors and radiomic characteristics between the two groups. The results of this study showed that one clinically independent risk factor and six radiomics features were associated with myocardial ischemia. This study compared clinical factors between the two groups, and showed that Gensini score was an independent predictor of myocardial ischemia, which were consistent with previous studies (21–23).

Radiomics is a method of transforming images to generate high-throughput data that can be mined. It has shown excellent performance in predicting the diagnosis, prognosis, staging, and treatment responses of cancer patients (24, 25). In this study, a predictive model was established based on CCTA radiomics. The AUC, sensitivity, specificity, accuracy, positive predictive, and negative predictive values of the model for predicting myocardial ischemia implying that the model has good diagnostic performance. To further enhance the performance of the model, this study combined clinical risk factors and radiomic features to establish a prediction model.

In this study, we developed a combined model incorporating CCTA and clinical variable to detect myocardial ischemia. Our results revealed that the combined DL model contributed to better performance for predicting myocardial ischemia with AUC values of 0.810 and 0.800 in the internal validation and external datasets. Hu and co-workers (26) evaluated 1,409 radiomics features from CTA images from patients with myocardial ischemia and constructed a logistic regression model that demonstrated AUC values of 0.762 and 0.671 for the training and test cohorts, respectively. In another study, Zhao et al. (27) extracted 385 radiomics features from target lesions on CTA images and constructed a logistic regression model, which demonstrated AUCs for predicting myocardial ischemia of 0.835 and 0.717 for the training and test cohorts, respectively. In the present study, the AUC values of the training set and internal validation set were 0.873 and 0.810, respectively, indicating that the prediction performance of our combined model was better than that of the model based only on image images.

As known that, the reproducibility and reliability of radiomics features were affected by the segmentation, as twice segmentation by same operator with different time, and by different operators were hardly exactly same, which caused radiomic features varied largely. But in our study, we used pre-trained VNET myocardium segmentation model to segment, which excluded the subjective deviation, and confirmed the segmentation at different time and with different operator were exactly same. This is a small advantage of this study.

Despite promising findings, our study has some limitations. Firstly, although the study included patients from two centers, the number of patients was still insufficient, only 110 patients were included in the study, which might cause overfitting, more data is needed in further to build a more robust model. However, we used independent internal validation dataset and external validation to validate the model, and found our model performed well in both two dataset, and their AUC values were close to AUC in training dataset, which decreased the overfitting suspicion. Nevertheless, in order to further validate the results of this study, the sample size should be further expanded in future multi-center studies. Secondly, retrospective studies may inevitably lead to selection bias. Finally, CCTA and clinical data alone are not perfect predictors of myocardial ischemia, and more valuable data, such as cardiac magnetic resonance, should be considered.

In conclusion, the diagnostic model based on CCTA radiomic features can predict myocardial ischemia. The combined diagnostic model with clinical risk score has higher clinical value, which provides more accurate diagnosis and treatment of myocardial ischemia.

## Data availability statement

The data analyzed in this study is subject to the following licenses/restrictions: With the authorization of the patient or his immediate family and through the decision of the Ethics Committee of Changzhou Second People’s Hospital paid by Nanjing Medical University. Requests to access these datasets should be directed to txq01040005@163.com.

## Author contributions

Y-CY: substantial contributions to conception and design and drafting the manuscript and critical revision for important intellectual content. YD, Z-WW, R-HY, C-JP, S-FD, and X-QT: data acquisition, data analysis, and interpretation. All authors contributed to final approval of the version to be published and agreed to be accountable for all aspects of the work to ensure that questions regarding the accuracy or integrity of the work were appropriately investigate and resolved.

## Footnotes

1
https://pyradiomics.readthedocs.io/en/latest/

2
https://cran.r-project.org/
